# Effects of forage inclusion and cattle breed on apparent digestibility and ruminal pH of steers fed a whole shelled corn-based diet

**DOI:** 10.1093/tas/txab221

**Published:** 2021-11-28

**Authors:** Pedro H V Carvalho, Mariana F Westphalen, Flavia A S Silva, Tara L Felix

**Affiliations:** Department of Animal Science, The Pennsylvania State University, University Park, PA 16802, USA

**Keywords:** Angus, cattle, digestibility, forage inclusion, Holstein, whole shelled corn

## Abstract

Objectives were to evaluate the effects of cattle breed, Holstein or Angus, and forage inclusion on total tract digestibility and ruminal pH in cattle fed a whole shelled corn-based diet. Six Holstein and six Angus steers were assigned to a 2 × 3 factorial arrangement of treatments. Factors included breed, Holstein or Angus, and forage inclusion at 0%, 8%, or 16% forage (dry matter [DM] basis). Steers were fed in a replicated 3 × 3 Latin square, split-plot design. Each period consisted of 14 d diet adaptation followed by 7 d of sample collection. Data were analyzed using the MIXED procedures in SAS (v9.4 SAS Inst. Inc., Cary, NC). Repeated measures were used to analyze changes in ruminal pH over time. There was no interaction of breed × diet (*P* ≥ 0.19) on dry matter intake (DMI) or digestibility; however, Holstein steers had greater (*P* = 0.03) DMI than Angus steers. Despite the impact of breed on intake, there was no effect (*P* ≥ 0.33) of breed on diet digestibility. Digestibility of DM increased (linear; *P* < 0.01) as forage was removed from the diet, but there were no differences (*P* ≥ 0.32) in Neutral Detergent Fiber (NDF) and starch digestibility. However, due to the change in diet, NDF intake digested on a grams per day basis increased (*P* ≤ 0.01) and starch intake digested (g/d) decreased (*P* = 0.01) as forage inclusion increased. There was a tendency for breed × diet interaction (*P* = 0.08) on ruminal pH. Holstein steers fed 8% or 16% forage had greater ruminal pH than Holstein steers fed 0% forage; but, ruminal pH of Angus steers was not altered by diet.

## INTRODUCTION

Changes in numbers of Holstein cattle being fed for beef production reported by (Boykin et al., 2017) have raised questions regarding best practices for feeding and managing Holstein steers in the feedlot. Fox et al. (1988) reported that Holstein steers have an 8% greater dry matter intake (DMI) than beef breeds during the feedlot phase. In addition, many Holsteins steers are fed a whole shelled corn (WSC)-based diet for ad libitum intake with little or no access to forage for the entire finishing period (Schaefer et al., 2017). According to Owens et al. (1997), WSC-based diets are often fed with less forage than the more typical dry-rolled corn (DRC)-based feedlot cattle diet. These authors suggested that decreased dietary forage inclusion when cattle are fed WSC might be the reason for the similar performance reported in native beef cattle fed WSC when compared to cattle fed DRC-based diets (Owens et al., 1997). Murphy et al. (1994) reported that cattle limit-fed a WSC-based diet with 0% forage had decreased total tract starch digestibility when compared to cattle fed a WSC-based diet for an ad libitum intake. The observed reduction in digestibility occurred when cattle were fed at 30% restriction (70% of the DMI that cattle fed for ad libitum intakes consumed) and the authors concluded that limiting cattle intake decreased the WSC particle size reduction caused by mastication when cattle had no access to forage (Murphy et al., 1994).

While Murphy et al. (1994) demonstrated the impact of altered intake in native beef cattle fed WSC with no forage, there is no information regarding how the potential greater DMI of Holsteins steers compared to Angus steers may impact total tract digestibility when cattle are fed WSC-based diets. Previous research has suggested that differences in diet digestibility among cattle breeds exist and that these differences may be due to changes in rumen microbial population among breeds altering the rate of feed digestion (Moore et al., 1975; Beecher et al., 2014). However, this research on breed differences involved diets with greater forage inclusion than what is typical in modern Holstein feedlot production systems. Thus, many recommendations for Holsteins entering the feedlot are being made with data from beef cattle that may or may not be applicable to Holsteins.

We hypothesized that Holstein steers would have greater DMI and, thus, greater diet digestibility when compared to Angus steers fed a WSC based-diet, and that the magnitude of this increase would be greater when cattle are fed a 0% forage diet. The objectives of this experiment were to evaluate the effects of cattle breed, Holstein or Angus, and forage inclusion on total tract digestibility and ruminal pH in cattle fed a WSC-based diet.

## MATERIALS AND METHODS

All procedures involving the use of animals were approved by The Pennsylvania State University Institutional Animal Care and Use Committee (#47255) and followed the guidelines recommended in the Guide for the Care and Use of Agricultural Animals in Research and Teaching (FASS, 2010).

### Animal and Diet Management

Six Holstein steers (body weight [BW] = 695 ± 5.8 kg; 30 ± 1 months of age) and six Angus steers (BW = 715 ± 7.6 kg; 30 ± 1 months of age), previously fitted with rumen cannulae, were assigned to a 2 × 3 factorial arrangement of treatments. The first factor was cattle breed, Holstein or Angus, and the second was forage inclusion, at 0%, 8%, or 16% forage inclusion (dry matter [DM] basis). Thus, the six treatments in the experiment were: 1) Holsteins fed 16% forage, 2) Holsteins fed 8% forage, 3) Holsteins fed 0% forage, 4) Angus fed 16% forage, 5) Angus fed 8% forage, and 6) Angus fed 0% forage. Diets were formulated to be isonitrogenous and meet the requirements of growing, British cattle breeds (NRC, 2000; Table 1).

**Table 1.** Composition of diets fed to Angus and Holstein steers

Item	Forage level		
	0	8	16
Ingredients, % DM basis			
Corn	90.0	81.8	73.5
Soybean meal	7.5	7.8	8.0
Grass hay	0.0	8.0	16.0
Supplement^1^	2.0	2.0	2.0
Urea	0.5	0.5	0.5
Analyzed nutrient composition			
DM	89.2	89.5	89.7
CP	13.1	12.9	12.6
NDF	10.4	14.5	18.7
Starch	69.2	63.3	57.4

^1^Mineral and vitamin supplement: 35.6% Urea, 1,550 g/ton Rumensin 90 (198 g of monensin/kg of DM; Elanco Animal Health, Greenfield, IN), Ca 25% (as CaSO_4_), NaCl 15%, Mg 1% (as MnSO_4_), K 3.5% (as KCl), Zn 1,000 mg/kg (as ZnSO_4_), Cu 180 mg/kg (as CuSO_4_), Se 16 mg/kg (as Na_2_SeO_3_), Vit A 286,600 IU/kg.

Feed was delivered once daily (0700) and steers were fed for ad libitum intakes (up to 0.25 kg of refusals) to restrict sorting of the diet. Steers were housed in individual metabolism stalls at the Beef Nutrition Research Lab, State College, PA. Stalls (2.5 × 1.5 m) were floored with rubber mats (Ani-mat Inc., Sherbrooke, QC, Canada) and equipped with individual feed bunks and non-siphoning automatic water bowls.

### Sampling and Analysis

Cattle were fed in a replicated 3 × 3 Latin square split-plot design. Each period was 21 d with 14 d for adaptation followed by 7 d of sample collection. On d 1 of each collection phase, ruminal pH was measured by collecting whole, mixed rumen contents via the rumen cannula at 0, 3, 6, 9, 12, and 18 h post morning feeding. Whole rumen content samples were strained through two layers of cheesecloth. The extracted liquid was immediately analyzed for pH using a FiveEasy FiveGo pH meter F20 with a LE438 polyoxymethylene body gel-filled electrode with Ag/AgCl reference system and 1.2m BNC/Cinch connection (Mettler Toledo, Columbus, OH). The pH meter was calibrated, according to manufacturer guidelines, before every sample collection.

Samples of individual feed ingredients (100 g/d as-is basis) and refusals (100 g sample/kg refusal as-is basis) were collected daily during the sample collection period. Feces were collected in canvas bags secured by a leather harness attached to the girth and under the neck of the steers. Feces were emptied from the bags, weighed, and subsampled (10% of the total feces as-is basis) twice daily over the 120-h collection phase (d 2 to 7). Feed ingredients, feed refusal, and fecal samples were composited within steer and period of collection and dried at 55 °C for 72 h. Dry, composited samples were ground through a Wiley mill (1-mm screen, Arthur H. Thomas, Philadelphia, PA). Ground samples were analyzed for NDF (using Ankom Technology method 6; Ankom200 Fiber Analyzer, Ankom Technology, Macedon, NY), N (using a Costech ECS 4010 C/N/S elemental analyzer; Costech Analytical Technologies Inc., Valencia, CA), starch and soluble sugars (determined by the method of Hall, 2009), and total ash (500 °C for 12 h, using a HotPack Muffle Oven Model: 770750, HotPack Corp., Philadelphia, PA). The resulting analyses of individual feed ingredients were used to calculate the nutrient composition of the diets (Table 1).

### Statistical Analysis

The experimental design was a replicated 3 × 3 Latin square split-plot design, where breeds represented the plots. Data were analyzed using the MIXED procedure of SAS (version 9.4; SAS Inst. Inc., Cary, NC). A Kenward–Roger adjustment was used. The model for DMI and apparent total tract digestibility was:


$$\begin{array}{ll} Y_{ijklmn}= & \;\mu +\;s_{i}+\;a_{j(i)}+\;p_{k(i)}+\;B_{l}\\ & +\;D_{m}+\;{\left(BD\right)}_{lm}+\;e_{ijklmn} \end{array}$$

in which Y_ijklmn_ is the response variable; μ is the mean; s_i_ = the random effect of square; *a*_j(i)_ = the random effect of animal nested within square; p_k(i)_ the random effect of period nested within square; B_l_ is the fixed effect of breed; D_m_ is the fixed effect of the diet; (BD)_lm_ is the interaction of breed × diet; e_ijklmn_ is the experimental error. When an interaction did not exist, orthogonal contrasts were used to test the linear and quadratic effect of forage inclusion. Means were separated using the LSMEANS statement with PDIFF option.

Repeated measures were used to analyze the effects of treatment on ruminal pH over time. The autoregressive heterogeneous, ARH(1), covariance structure was chosen based on the smallest Bayesian Information Criterion. A Kenward–Roger adjustment was used. The model was:


$$\begin{array}{ll} Y_{ijklmno}= & \;\mu +\;s_{i}+\;a_{j(i)}+\;p_{k(i)}+\;B_{l}+\;D_{m}+\;{\left(BD\right)}_{lm}+\;T_{n}\\ & +\;{\left(BT\right)}_{ln}+\;{\left(DT\right)}_{mn}+\;{\left(BDT\right)}_{lmn}+\;e_{ijklmno} \end{array}$$

in which Y_ijklmno_ is the response variable; μ is the mean; s_i_ = the random effect of square; a_j(i)_ = the random effect of animal nested within square; p_k(i)_ the random effect of period nested within square; B_l_ is the fixed effect of breed; D_m_ is the fixed effect of the diet; (BD)_lm_ is the interaction of breed × diet; T_n_ is the fixed effect of time of collection; (BT)_ln_ is the fixed effect of the interaction of breed × time of collection; (DT)_mn_ is the fixed effect of the interaction of diet × time of collection; (BDT)_lmn_ is the fixed effect of the interaction of breed × diet × time of collection; e_ijklmno_ is the experimental error. Means were separated using the LSMEANS statement with PDIFF option. Individual steer within period was the experimental unit.

Significance was declared at *P* ≤ 0.05. Trends are discussed at 0.05 < *P* < 0.10.

## RESULTS AND DISCUSSION

The 2 × 3 factorial arrangement was established to evaluate the effects of cattle breed, Holstein or Angus, and forage inclusion on total tract digestibility and ruminal pH in cattle fed a WSC based diet. However, there were no breed × diet interactions (*P* ≥ 0.19) on DMI, intake of digestible nutrients, or diet digestibility (Table 2). Due to the increasing relevance of the Holstein breed in feedlot systems (Boykin et al., 2017), the main effects of these responses are provided and discussed.

**Table 2.** Effects of cattle breed, Holstein or Angus, and forage inclusion on diet intake and digestibility, and nutrient digestible intake

Item, DM basis	Diet^1^			Breed			*P* 2			
	0	8	16	Angus	Holstein	SEM	Diet		B	D × B
							Linear	Quad		
*n*, animals	12	12	12	6	6					
Calculated DM Intake, % of BW^3^	1.85	1.93	1.97	1.83	2.00	0.085	0.21	0.83	0.03	0.65
Intake, kg/d^3^										
DM	13.06	13.55	13.76	13.04	13.87	0.453	0.24	0.78	0.09	0.63
OM	12.01	12.47	12.66	11.99	12.76	0.417	0.24	0.78	0.09	0.63
NDF	1.29	1.81	2.37	1.77	1.87	0.058	<0.01	0.83	0.12	0.19
Starch	8.32	7.89	7.26	7.59	8.06	0.270	0.01	0.73	0.11	0.69
Digestibility, %^4^										
DM	79.1	74.2	72.5	74.8	75.7	1.03	<0.01	0.22	0.46	0.81
OM	78.6	73.8	72.3	74.4	75.4	1.11	<0.01	0.25	0.47	0.83
NDF	51.4	52.9	54.4	51.4	54.4	2.74	0.32	0.99	0.33	0.94
Starch	90.4	89.1	89.3	89.3	89.9	0.89	0.42	0.51	0.62	0.72
Digestible Intake, kg/d^5^										
DM	10.36	10.05	9.97	9.76	10.50	0.386	0.48	0.81	0.11	0.85
OM	9.48	9.20	9.15	8.94	9.61	0.354	0.52	0.80	0.11	0.87
NDF	0.66	0.97	1.30	0.92	1.03	0.055	<0.01	0.80	0.07	0.32
Starch	7.52	7.03	6.48	6.78	7.23	0.241	0.01	0.93	0.11	0.83

^1^0 = 0% forage inclusion in the diet; 8 = 8% forage inclusion in the diet; 16 =16% forage inclusion in the diet.

^2^Linear = Linear effect of diet; Quad = Quadratic effect of diet; B = effect of breed; D × B = interaction of diet × breed.

^3^Calculated as kg of nutrient consumed per day on DM basis.

^4^Calculated as g of nutrient digested per kg of total diet consumed on DM basis.

^5^Calculated as kg of nutrient digested per day on DM basis.

As forage inclusion increased in the diet, total tract DM and OM digestibility decreased (Linear; *P ≤* 0.01; Table 2). However, there were no differences (*P* ≥ 0.32) in total tract NDF and starch digestibility (% on DM basis) among dietary treatments. Although total tract digestibility (% on DM basis) of NDF and starch were similar among dietary treatments, changes in NDF and starch intake (kg/d) affected (Linear; *P ≤* 0.01) NDF and starch digested on a grams per day basis. As forage inclusion in the diet increased, the kilograms per day of NDF that was digested increased and the kilograms per day of starch digested decreased. These changes are not uncommon with forage inclusion trials in feedlot cattle and were to be expected.

Perhaps more interesting to the parameters of the current study was the main effect of breed (*P* = 0.03) on DMI (% of BW; Table 2). On average, Holstein steers had a 9% greater DMI than Angus steers as a percent of BW (2.00 vs. 1.83 kg DMI/% of BW, respectively), but not in kg/day. It had been hypothesized that Holstein cattle would consume more than Angus, due to the differential selection of those two breeds. For many years, Holsteins have been selected for increasing DMI as DMI drives milk production, and it is known that Holstein steers in the feedlot consume more than native beef breeds (Fox et al., 1988). Meanwhile, a greater emphasis has been placed on the efficiency of feed conversion in the Angus breed. Despite the hypothesis that digestibility may be altered relative to the expected increasing DMI, there was no main effect of breed (*P* ≥ 0.33) on total tract DM, OM, NDF, or starch digestibility (g/kg). In addition, there was not a main effect (*P* = 0.11) of breed on kg of DM, OM, and starch intake digested per day. Generally, changes in intake impact digestibility result in cattle diets (Murphy et al., 1994). However, the only difference noted in this study was that Holstein steers tended (*P* = 0.07) to digest 12% more NDF intake than Angus steers on a daily basis.

A similar increase in DMI of Holstein steers compared to Angus without negative effects on DM digestibility (DMD) has been reported by Tjardes et al. (2002) when cattle were fed a corn-silage based diet. These authors reported that Holstein steers had a 14% greater DMI than Angus steers with no breed effect on DMD (Tjardes et al., 2002). However, Tjardes et al. (2002) and Carvalho et al. (2020) reported that Angus steers had greater NDF digestibility (NDFD) than Holstein steers when cattle were fed 20% or more forage (DM basis) in their diets. Similarly, Murphy et al. (1994) stated that cattle fed WSC had greater diet digestibility when DMI was increased. Murphy et al. (1994) hypothesized that this impact of DMI on digestibility occurred because cattle that ate more reduced the particle size of the corn kernel better than cattle that ate less. However, even though Holstein steers had greater intake than Angus steers, there was no effect of breed on diet digestibility of cattle fed WSC based diet. This may be due to the greater magnitude of change in the Murphy et al. (1994) work due to the restricted, or programmed, feeding of cattle in that study versus the relatively small changes in intake between Angus and Holstein cattle current experiment.

As anticipated, regardless of breed, as forage inclusion in the diet increased, DMD and OMD were reduced (Table 2). Increased DMD observed in cattle fed all concentrate (0% forage) diets may be related to the increased rumen microbial fermentation expected when cattle are fed grains compared to forage (Dixon and Stockdale, 1999). Increasing forage concentration in the diet greatly increased the NDF composition of the diet, and subsequent NDF intake (kg/d). However, an inverse relationship is observed on the dietary starch concentration and starch intake, as forage is added into the diet, starch concentration and starch intake decrease (kg/d). De Souza et al. (2018) reviewed data of 54 diet digestibility studies from high producing lactating dairy cows and reported a positive relationship between NDF concentration in the diet and total tract NDFD. Moreover, these authors also reported that as the starch concentration in the diet increases, total tract NDFD decreases (De Souza et al., 2018). However, the maximum inclusion of forage fed to the beef cattle in the current experiment was 16% of the diet (DM basis), whereas De Souza et al. (2018) fed 43% of the diet as forage (DM basis) at the lowest inclusion. The current experiment has no effect of forage (NDF) inclusion on total tract NDF and starch digestibility. As noted previously, while there were no changes in NDFD (g/kg) among treatments, the kg per day of NDF intake that was digested greatly changed among cattle fed 0%, 8%, and 16% forage. However, these changes in NDF, and starch, digested per day are more likely caused by the changes in nutrient intake and not by the rate of nutrient digestion.

**Figure 1.**
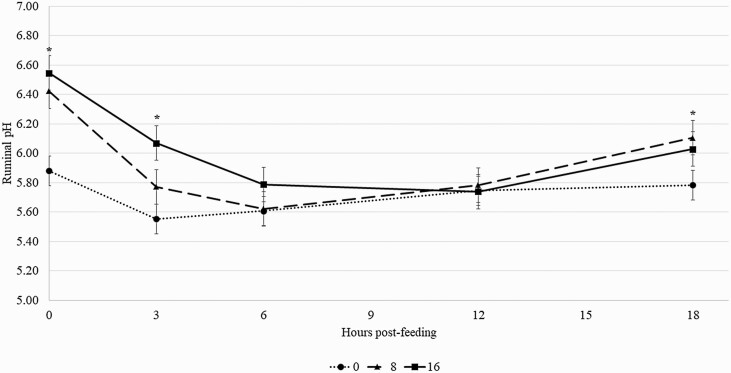
Effects of forage inclusion on ruminal pH over time. Black solid line (●     ) = Steers fed 16% forage (% DM basis); black dashed line (■) = Steers fed 8% forage (% DM basis); black dotted line (▲) = Steers fed 0% forage (% DM basis). There was a diet × hour interaction on ruminal pH (*P* < 0.01). The use of (*) denotes differences (*P* < 0.05) between breeds within hour. Error bars are associated with the interaction between diet × hour (SEM = 0.1171).

There was no breed × diet × hour interactions (*P* = 0.88) on ruminal pH. However, there was a diet × hour interaction (*P* < 0.01) on ruminal pH (Figure 1). Regardless of breed, steers fed the 0% forage diet had the most acidic ruminal pH at 0, 3, and 18 h post-feeding, with no effect of diet on ruminal pH at 6 and 12 h post-feeding. Steers fed 8% forage, and 16% forage had similar ruminal pH over time, except at 3 h post-feeding when steers fed 16% forage had greater ruminal pH than steers fed 8% forage.

**Figure 2.**
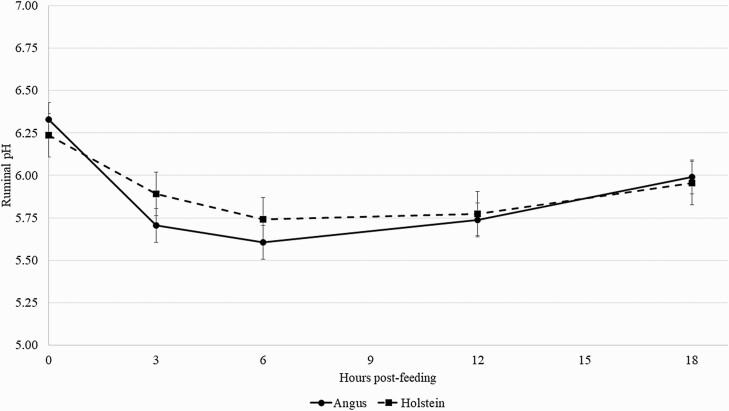
Effects of cattle breed on ruminal pH over time. Black solid line (●    ) = Angus steers; black dashed line (■) = Holstein steers. There was an interaction between breed × hour (*P* = 0.05). Error bars are associated with the interaction between breed × hour (SEM = 0.1289).

**Figure 3.**
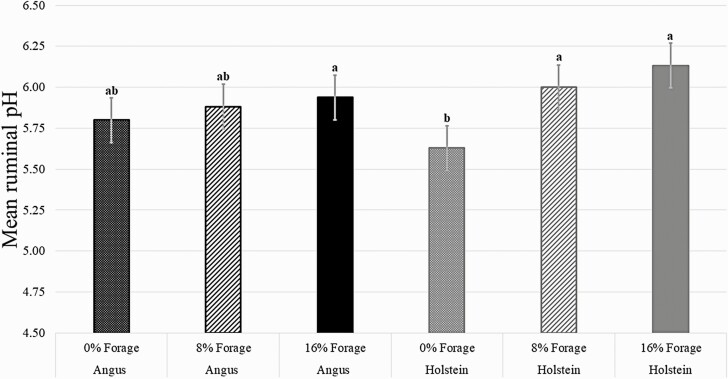
Effects of cattle breed, Holstein or Angus, and forage inclusion on mean ruminal pH. Black dotted bar = Angus steers fed 0% forage (% DM basis); black dashed bar = Angus steers fed 8% forage (% DM basis); black solid bar = Angus steers fed 16% forage (% DM basis); gray dots line = Holstein steers fed 0% forage (% DM basis); gray dashed bar **=** Holstein steers fed 8% forage (% DM basis); gray solid bar = Holstein steers fed 16% forage (% DM basis). There was no breed × diet × hour (*P* = 0.88). However, there were breed × hour (*P* = 0.05) and diet × hour (*P* < 0.01) interactions on ruminal pH, and a tendency for breed × hour (*P* = 0.08) interaction. Different superscripts (a, b) denote differences (*P* ≤ 0.05) among treatments. Error bars are associated with the interaction between diet × hour (SEM = 0.1365).

There was a breed × hour (*P* = 0.05) interaction for ruminal pH. Regardless of diet, Angus steers had a greater decrease in ruminal pH from 0 to 3 h post-feeding than Holstein steers (Figure 2). Ruminal pH of both breeds decreased in a similar magnitude from 3 to 6 h hours post-feeding and increased from 6 to 12 h post-feeding. Ruminal pH did not differ between the two breeds at 12 and 18 h post-feeding. In addition, there was a tendency for a breed × diet (*P =* 0.08) interaction on mean ruminal pH (Figure 3). As forage inclusion increased in the diet, the mean ruminal pH of Angus steers did not change. However, the mean ruminal pH of Holstein steers fed 0% forage was less than the mean ruminal pH of Holstein steers fed either 8% or 16% forage in their diets.

Regardless of breed, cattle fed the 0% forage diet maintained the most acidic ruminal pH over time (Figure 1. Moreover, cattle fed the 0% forage diet had ruminal pH below 5.8 in 4 of the 5 time points measured in the current experiment. As previously mentioned, this results from greater rumen fermentation of grains in the rumen (Dixon and Stockdale, 1999). According to Nagaraja and Titgemeyer (2007) and Owens et al. (1998), a steer adapted to a grain-based diet with a healthy and functional rumen would have a ruminal pH that should vary from 5.8 to 6.5. As ruminal pH starts to fall below this range, cattle may start to experience a subclinical (subacute) acidosis with detrimental effects on DMI, feed digestion, and subsequent animal performance (Owens et al., 1998; Nagaraja and Titgemeyer, 2007). In the current experiment, even though steers fed 0% forage were suffering from a potential subclinical acidosis, there was no dietary effect on DMI and diet digestibility. However, it is important to emphasize that steers in the current experiment were fed each diet for no longer than 21 d period; therefore, the potential long-term negative effect of feeding animals a 0% forage-based diet cannot be extrapolated with data from the current experiment.

The interaction between breed × hour on ruminal pH over time reflects the greater decrease observed in ruminal pH from 0 to 3 hours post-feeding of Angus steers compared to Holstein steers (Figure 2). Carvalho et al. (2020) limit-fed Angus and Holstein steers either a 20% or 80% forage-based diets, and concluded that regardless of diet, Holstein steers spent more time ruminating their diet than Angus steers; therefore, Holstein steers had the greater potential of buffering ruminal pH than Angus steers. In the current experiment, Holstein steers had 9% greater DMI (% of BW) and 11% greater starch intake (kg/d) than Angus steers, but Holstein steers were able to maintain a similar or greater ruminal pH than Angus steers throughout the day. However, the tendency on the interaction between breed × diet (Figure 3) suggests that this greater buffering capacity of Holstein steers could hold true only when a source of forage is provided in the diet. Holstein steers fed 0% forage had a greater decrease in mean ruminal pH than Angus steers fed 0% forage compared to steers fed 8% or 16% forage in their respective breeds. More research is needed to determine the interaction between cattle breed and forage inclusion in the diet on ruminal pH and how these data, when feeding long-stem hay, may be extrapolated to cattle fed corn silage as a forage source as well.

It was hypothesized that Holstein steers fed a WSC-based diet would consume more DM and, as a result, have greater digestibility when compared to Angus steers and that the magnitude of this response would be greater when forage was removed from the diet. Holstein steers consumed 9% more DM (% of BW) than Angus steers; however, there was no interaction between breed × diet or main effect of breed on total tract diet digestibility. Decreasing forage in the diet did decrease the amount of NDF intake digested daily but increased the amount of starch intake digested daily. Despite these changes in DMI relative to Angus steers, Holstein steers maintained similar or more alkaline ruminal pH compared to Angus steers throughout the day. Regardless of breed, steers fed a 0% forage diet were experiencing a ruminal pH consistently below 5.8, which may have long-term negative effects on animal performance.
